# Exploring association between food insecurity and depression among older adults in India

**DOI:** 10.1016/j.dialog.2022.100042

**Published:** 2022-08-27

**Authors:** Ratna Patel, Shubham Kumar, Shekhar Chauhan

**Affiliations:** a Department of Public Health and Mortality Studies, International Institute for Population Sciences, Mumbai, India; b Department of Mathematical Demography & Statistics, International Institute for Population Sciences, Mumbai, India; c Department of Family and Generations, International Institute for Population Sciences, Mumbai, India

**Keywords:** Food insecurity, Depression, Older adults, India

## Abstract

**Background:**

Depression is a significant health concern that is yet to be recognised as an important public health challenge in India. Furthermore, given the critical condition of food insecurity among older people in India, it is likely that they are more vulnerable to depression. The interplay of depression among older people resulting from food insecurity is an under-explored phenomenon in the Indian context. Therefore, this study examines the association between food insecurity and depression among older people in India.

**Methods:**

The study used data from the Longitudinal Ageing Study in India (LASI). Food insecurity was measured with a set of questions formed into dichotomous variable and depression was measured with Center for Epidemiological Studies Depression (CES-D scale). Binary logistic regression was performed to confirm the findings.

**Results:**

Results showed that older adults who reported food insecurity were more likely to be depressed (OR= 1.20; C.I.=1.03-1.25) than their younger counterparts. Furthermore, older adults who were independent for Activity of Daily Living (ADL) were less likely (OR= 0.73; C.I.=0.53-1.00) to report depression, whereas, female (OR= 1.12; C.I.=1.00-1.26) and never married (OR= 2.11; C.I.=1.18-3.79) older adults were more likely to be depressed than their respective counterparts.

**Conclusion:**

It is important to integrate mental health with food insecurity. Future studies may consider including mental health services with food assistance programs or vice versa.

## Highlights

• Higher likelihood of depression was noted among those having food insecurity.
• Higher likelihood of depression was also highlighted among those not independent for ADL and IADL.
• It is important to integrate mental health with food insecurity.

## 1 Introduction:

Food insecurity is a condition of limited access to safe and healthy food [18]. In contrast, food security refers to a situation when ‘all people’ at all times have physical, social, and economic access to sufficient, safe, and nutritious food that meets their dietary needs and food preferences for active and healthy life [17]. Despite the decline in the overall global rates of food insecurity since the early 1990s, there remained a widespread inequality with higher undernourishment rates recorded in Sub-Saharan African, Caribbean, and South-Asian countries [18]. The inequality in food insecurity is also prevalent in India [13,43]. Food insecurity has been associated with several chronic illnesses, including diabetes and hypertension [7,8,45], obesity [45], and asthma [20,45]. Furthermore, food insecurity has also been linked to psychological well-being [22]. Adverse mental health outcomes linked to food insecurity are of increasing concern [14,21,24,29].

Depression is a significant health concern that is yet to be recognised as an important public health challenge in India [36,39]. Furthermore, depression becomes the most common mental health problem in late life [33]. The older population in India is growing three times faster than the overall population [19]. Given the rising proportion of India’s aged population [10,37], depression among older people is a topic worth exploring [38]. Furthermore, given the critical condition of food insecurity among older people in India [9], it is likely that they are more vulnerable to depression. The interplay of depression among older people resulting from food insecurity is an under-explored phenomenon in Indian context.

Depression is a growing concern, and a growing body of research has demonstrated a link between food insecurity and depression [12,[23], [24], [25]]. However, the relation between food insecurity and mental health has been explored in a few studies with mixed findings [22]. Literature examining the link between food insecurity and depression among children [31] and adults [6,12,23,25] is widely available; however, the same literature is missing for older adults in India [42]. The relationship between food insecurity and depression concerns in light of the high rates of depression among older people [38,39]. Therefore, this study examines the association between food insecurity and depression among older people in India by utilizing information from the recently released Longitudinal Ageing Study Survey, Wave-I (LASI) in India.

## 2 Materials and methods

### 2.1 Data

The data has been drawn from the first wave of the Longitudinal Ageing Study in India (LASI), which was carried out in 2017-18 for adults 45 years and older [27]. The LASI is a multidisciplinary and internationally harmonized panel study of 72 250 older adults, including 31,464 elderly aged 60 years and above and 6,749 oldest-old persons aged 75 years and above from 35 states and union territories (UTs) of India (except Sikkim). The LASI is the world’s largest and India’s first-ever longitudinal survey which evaluated the effect of changing policies on the behavioural outcomes for older adults in India

The LASI is conceptualized to provide comprehensive knowledge of key health outcomes and the social and economic well-being of the elderly. The key objective of the survey is to provide a shred of scientific evidence on demographics, household economic status, chronic health conditions, symptom-based health conditions, functional health, mental health (cognition and depression), biomarkers, health insurance, and healthcare utilization, family and social networks, social welfare programmes, work and employment, retirement, satisfaction, and life expectations. The survey was funded by the Ministry of Health and Family Welfare (MoHFW), the Government of India, the National Institute on Aging (NIA), and the United Nations Population Fund, India (UNFPA). In addition, three nodal agencies were assigned for this survey as International Institute for Population Sciences (IIPS), Harvard T.H. Chan School of Public Health (HSPH), and the University of Southern California (USC).

The LASI has been used a multistage sampling design to select the representative sample across states and UTs in India. Within each state and UTs, a three-stage sampling design was adopted in rural areas and four stage-sampling techniques for urban areas. In the first sampling stage, primary sampling units (PSUs) were selected, Tehsils and Talukas for rural and urban areas. The second stage involved selecting villages in rural areas and wards in urban areas in selected PSUs. In the third stage, households were selected in rural areas, and one census enumeration block (CEB) was randomly chosen in each ward in urban areas. Finally, in the fourth stage of sampling, households were selected from selected CEBs.

### 2.2 Food insecurity variable

The questionnaire included a series of questions about food security concerns in the last 12 months because of limited food availability (for example, In the last 12 months, did you ever reduce the size of your meals or skip meals because there was not enough food at your household?).

#### 2.2.1 Household food insecurity classification

The calculation of household food insecurity score was adopted from FANTA (Food and Nutrition Technical Assistance) which provide household food insecurity access scale (HFIAS). In addition, FANTA conducted the study Tufts University and Cornell university to review and further validate the scale. Likewise, LASI has also adopted the household food insecurity scale in a same way which includes following question:

• In the last 12 months, did you ever reduce the size of your meals or skip meals because there was not enough food at your household?
• In the last 12 months, did you eat enough food of your choice? Please exclude fasting/food related restrictions due to religious or health related reason.
• In the last 12 months, were you hungry but didn’t eat because there was not enough food at your household? Please exclude fasting/food related restrictions due to religious or health related reasons.
• In the past 12 months did you ever not eat for a whole day because there was not enough food at your household? Please exclude fasting/food related restrictions due to religious or health related reasons.
• Do you think that you have lost weight in the last 12 months because there was not enough food at your household?
  All the questions collected the response either “yes” or “no”. In addition, the respondents answered questions about whether the certain situation had occurred in the last 12 months because of having limited food availability. For analysis, the responses were aggregated into “food secure” and “food insecure.” These questions were drafted keeping in mind the Indian scenario. Policy discussions are going on in the country to ensure food and nutrition security for the people, thereby drafting these questions might help the policy discourse in the country.

### 2.3 Center for epidemiological studies depression (CES-D)

Andersen and group developed the center for epidemiological studies depression scale (CES-D-10), as well as the shorten version in 1994 [3]. The CES-D-10 included ten questions, each item has the same numerical rating scale, 1 represents “rarely or never (less than 1 day)”, 2 means “sometimes (1 or 2 days)”, 3 represents “Often (3 or 4 days)”, and 4 represents “most or all of the time (5-7 days)”. Total CES-D-10 scores can range from 10 to 40, and the cut-off scores for depressive symptoms were 16 or higher [3]. This scale measures number of days a person felt depressive symptoms in last one week, so this scale captures depressive symptoms for the last seven days.

### 2.4 Study variables

#### 2.4.1 Response variables

The response variables for this study are food security and CES-D-10. Further, food security has been classified into ‘food secure’ and ‘food insecure.’ Similarly, CES-D has been categorized into two categories as “yes” and “no.” We have categorized depression as outcome variable also as we have explored the factors affecting depression, with food insecurity being the important factor for which we intend to explore the association.

#### 2.4.2 Independent variables

The independent variables for this study are depressive symptoms (depressed and not depressed) – this was categorized using CES-D-10 scale; self-rated health (poor and good); rigorous physical activity (yes and no); Activity of daily Living (ADL) disability (severe. Moderate and no ADL disability), and Instrumental Activity of daily Living (IADL) disability (Severe, moderate and no IADL disability); sex (male and female); age (60-69 and 70 years and above); marital status (currently married, never married, Divorced/Separated/Deserted/Widowhood); education (No education, below primary, primary, secondary, and higher); living arrangements (living alone, with spouse and with others); place of residence (rural and urban); currently working (yes and no); wealth index (poorest, poorer, middle, richer and richest) and region (north, central, east, north-east, west and south). Variables including ADL, IADL, and wealth index were created using relevant information from the LASI data. ADL and IADL were created using a set of questions based on Katz model [26] and Lawton and Brody model [28] of ADL and IADL, respectively.

### 2.5 Statistical analysis

Descriptive statistics were used to summarize the proportion of food security, food insecurity, and CES-D by depression, health, and sociodemographic and economic characteristics of the elderly. Binary logistic regression was performed to estimate the adjusted association between food insecurity and depressive symptoms, health (Self-rated health, rigorous physical activities, ADL and IADL disability), and sociodemographic (sex, age, marital status, education, living arrangements, place of residence and region) and economic (currently working and wealth index) characteristics. Similarly, binary logistic regression was performed to estimate the adjusted association between CES-D and health and sociodemographic and economic characteristics. Further, to see the adjusted effect, we divided our logistic results in two segment as model I & II. Model I included only depression and health status variables where model II included depression and health status along with socio-demographic and economic variables. We have taken CES-D in various models separately to understand the effect of food insecurity and other interdependent variables on depression.

## 3 Results

Table 1 depicts the sociodemographic, health, and economic characteristics of the study sample. The study is based on 31,464 older people. Almost two-thirds (67.6%) of the elderly reported depression on the CES-D scale.

**Table 1:** Sociodemographic, health and economic characteristics of study sample in LASI survey from 2017-18.

Variables	N	%
**Depression and health status**		
***Depressive symptoms***		
Not depressed	9,843	32.4
Depressed	20,546	67.6
***Self-rated health***		
Poor	4,630	15.0
Good	26,181	85.0
***Rigorous physical activities***		
Yes	9,704	31.1
No	21,494	68.9
***ADL disability***		
Severe ADL	999	3.2
Moderate ADL	6,045	19.3
No ADL	24,291	77.5
***IADL disability***		
Severe IADL	1,859	5.9
Moderate IADL	13,281	42.4
No IADL	16,164	51.6
**Socio-demographic and economic**		
***Sex***		
Male	14,931	47.5
Female	16,533	52.6
***Age***		
60-69	18,410	58.5
70+	13,054	41.5
***Marital Status***		
Currently married	19,536	62.1
Never Married	225	0.7
Divorced/Separated/Deserted	11,703	37.2
***Education***		
No education	17,782	56.5
Below primary	3,598	11.4
Primary	3,520	11.2
Secondary	5,285	16.8
Higher	1,278	4.1
***Living arrangements***		
Living alone	1,787	5.7
With spouse	19,176	60.9
With others	10,501	33.4
***Place of residence***		
Rural	22,196	70.6
Urban	9,268	29.5
***Currently working***		
Yes	9,483	41.8
No	13,197	58.2
***Wealth Index***		
Poorest	6,829	21.7
Poorer	6,831	21.7
Middle	6,590	21.0
Richer	6,038	19.2
Richest	5,175	16.5
**Region**		
North	3,960	12.6
Central	6,593	21.0
East	7,439	23.6
North-east	935	3.0
West	5,401	17.2
South	7,136	22.7
**Total**	**31,464**	**100**

Table 2 depicts the bivariate association between depression and other background characteristics and food security and food insecurity. The results noted a higher depression level among the elderly facing food insecurity. The result noted that almost 53.3 percent of the elderly population with food security reported depression, whereas around 54.7 percent of the elderly population with food security were not depressed. Similarly, around 45.3 percent of the elderly with food insecurity were not depressed, whereas around 46.7 of the elderly with food insecurity were categorized as depressed on the CES-D scale.

**Table 2:** Bivariate description of food insecurity for depression and other study variables for LASI survey, 2017-18.

	Food security (%)	Food insecurity (%0	CES-D (%)	Total (N)
**Depression and health status**				
***Depressive symptoms***				
Not depressed	54.7	45.3	-	-
Depressed	53.3	46.7	-	-
***Self-rated health***				
Poor	46.8	53.2	73.8	4,567
Good	55.3	44.7	66.5	25,815
***Rigorous physical activities***				
Yes	56.1	43.9	68.6	9,583
No	52.8	47.2	67.2	20,787
***ADL disability***				
Severe ADL	40.8	59.2	74.1	762
Moderate ADL	52.0	48.0	75.0	5,779
No ADL	55.0	45.0	65.6	23,843
***IADL disability***				
Severe IADL	47.5	52.5	70.6	1,532
Moderate IADL	53.7	46.3	73.3	12,908
No IADL	54.9	45.1	62.7	15,912
**Socio-demographic and economic**				
***Sex***				
Male	55.4	44.7	66.6	14,332
Female	53.4	46.6	68.5	16,057
***Age***				
60-69	54.6	45.5	67.2	18,083
70+	54.0	46.0	68.2	12,306
***Marital Status***				
Currently married	55.7	44.3	66.5	18,907
Never Married	43.1	56.9	77.2	209
Divorced/Separated/Deserted	52.2	47.8	69.4	11,273
***Education***				
No education	51.2	48.8	66.0	17,150
Below primary	51.7	48.3	70.5	3,517
Primary	57.8	42.3	68.3	3,412
Secondary	61.9	38.1	69.8	5,060
Higher	63.5	36.5	70.8	1,248
***Living arrangements***				
Living alone	44.9	55.1	73.7	1,734
With spouse	56.1	43.9	66.5	18,585
With others	52.7	47.3	68.7	10,070
***Place of residence***				
Rural	52.2	47.8	66.5	21,602
Urban	59.4	40.6	70.3	8,787
***Currently working***				
Yes	54.3	45.7	65.4	9,377
No	55.0	45.0	70.0	12,527
***Wealth Index***				
Poorest	47.6	52.4	68.6	6,627
Poorer	54.0	46.0	65.9	6,618
Middle	56.8	43.2	65.4	6,307
Richer	57.7	42.3	67.6	5,839
Richest	56.4	43.6	71.4	4,999
**Region**				
North	62.0	38.0	62.1	3,895
Central	48.5	51.5	66.9	6,349
East	53.2	46.8	64.0	7,247
North-east	47.8	52.2	63.1	911
West	63.2	36.8	72.1	5,247
South	50.7	49.3	72.6	6,740
**Total**	**54.3**	**45.7**	**67.6**	**30,389**

Table 3 depicts the result for binary logistic regression. Results found that older adults who reported food insecurity were more likely to be depressed (OR= 1.20; C.I.=1.03-1.25) than their counterparts who were not food insecure. The elderly with good self-rated health were less likely (OR = 0.78; C.I. = 0.68-0.88) to be depressed. Similarly, elderly who were independent for ADL were less likely (OR = 0.73; C.I. = 0.53-1.00) to be depressed. Female (OR = 1.12; C.I. = 1.00-1.26), never married (OR= 2.11; C.I. = 1.18-3.79), and not currently working (OR = 1.05; C.I. = 1.08–1.34) were more likely to be depressed than their respective counterparts from male, currently married, and currently working categories. However, the elderly with higher education had a lower likelihood of food insecurity (OR = 0.62; C.I. = 0.51-0.93); however, they had higher odds of depression (OR = 1.39; C.I. = 1.09–1.77) than their counterparts.

**Table 3:** Odds ratio estimates showing association between food insecurity and depression by various background characteristics for LASI survey, 2017–18.

	Food insecurity		CES-D	
	OR	CI at 95%	OR	CI at 95%
**Model I**				
**Depression and health status**				
***Depressive symptoms***				
Not depressed®				
Depressed	1.20**	1.03-1.25		
***Self-rated health***				
Poor®				
Good	0.75***	0.67-0.84	0.78***	0.68-0.88
***Rigorous physical activities***				
Yes®				
No	1.10**	1.00-1.21	0.85***	0.78-0.94
***ADL disability***				
Severe ADL®				
Moderate ADL	0.61***	0.46-0.81	0.97	0.71-1.33
No ADL	0.56***	0.42-0.74	0.73**	0.53-1.00
***IADL disability***				
Severe IADL®				
Moderate IADL	1.01	0.81-1.24	1.37**	1.07-1.77
No IADL	1.03	0.82-1.27	0.92	0.70-1.18

**Model II**				
**Depression and health status**				
***Depressive symptoms***				
Not depressed®				
Depressed	1.05**	0.95-1.15		
***Self-rated health***				
Poor®				
Good	0.72***	0.63-0.81	0.73***	0.68-0.84
***Rigorous physical activities***				
Yes®				
No	1.12**	1.02-1.27	0.78***	0.70-0.87
***ADL disability***				
Severe ADL®				
Moderate ADL	0.70**	0.50-0.96	1.14	0.78-1.66
No ADL	0.62***	0.44-0.85	0.76	0.52-1.10
***IADL disability***				
Severe IADL®				
Moderate IADL	0.93	0.73-1.21	1.35**	1.01-1.81
No IADL	1.01	0.79-1.35	0.97	0.72-1.31
**Socio-demographic and economic**				
***Sex***				
Male®				
Female	1.00	0.89-1.11	1.12**	1.00-1.26
***Age***				
60-69®				
70+	1.02	0.92-1.12	0.94	0.84-1.04
***Marital Status***				
Currently married®				
Never Married	0.96	0.55-1.64	2.11**	1.18-3.79
Divorced/Separated/Deserted	0.54***	0.38-0.77	1.28	0.87-1.87
***Education***				
No education®				
Below primary	1.05	0.92-1.23	1.26*	1.06-1.49
Primary	0.78*	0.69-0.94	1.22**	1.04-1.44
Secondary	0.66***	0.61-0.80	1.25***	1.08-1.45
Higher	0.62**	0.51-0.93	1.39*	1.09-1.77
***Living arrangements***				
Living alone®				
With spouse	0.41***	0.28-0.61	1.07	0.70-1.62
With others	0.73***	0.61-0.91	0.87	0.70-1.08
***Place of residence***				
Rural®				
Urban	0.83***	0.73-0.93	1.06	0.93-1.18
***Currently working***				
Yes®				
No	0.90**	0.81-1.00	1.05***	1.08-1.34
***Wealth Index***				
Poorest®				
Poorer	0.78***	0.69-0.89	0.92	0.79-1.05
Middle	0.70***	0.61-0.81	0.89	0.77-1.03
Richer	0.70***	0.67-0.81	0.92	0.79-1.07
Richest	0.79***	0.67-0.92	1.07	0.91-1.24
**Region**				
North®				
Central	1.55***	1.35-1.79	1.50***	1.30-1.73
East	1.25***	1.10-1.42	1.18**	1.04-1.36
North-east	1.71***	1.46-2.01	1.28***	1.09-1.50
West	0.90	0.77-1.05	1.63***	1.39-1.91
South	1.62***	141-1.86	1.67***	1.46-1.91

®-Reference category.

## 4 Discussion:

This study found evidence of a relationship between food insecurity and depression among the elderly; however, the relationship might be misleading due to cross-sectional data where possible reverse causation can be a cause of concern. This study very well confirmed that food insecurity indeed has a positive association with depression when depression is measured with the CES-D scale. Several previous studies have noted this inference where food insecurity was noted as a predictor of depression among various sub-populations in different settings [6,23,29,41]. Huddleston-Casas [23] noted evidence for the relationship between food insecurity and depression in a sample of rural, low-income women in the USA using longitudinal data [23]. They concluded that the relationship between the two was recursive [23]. Silverman et al. [41] also noted a positive association between food insecurity and depression; however, they also examined diabetes distress, medication adherence, and glycemic control as other significant predictors along with depression [41]. The study by Leung et al. [29], focusing on 3518 adults in the US and assessing depression by using a 9-item Patient Health Questionnaire (PHQ) scale, also noticed a positive association between food insecurity and depression [29].

The authors failed to find any associative study in the Indian context examining the relationship between food insecurity and depression among the older population using a large sample. However, few studies in the Indian context also attempted to examine the association between food insecurity and depression, albeit with a different approach and differing population in various sub-settings [22,40]. Heylen et al. [22] examined the link between food insecurity and depression among people living with HIV in a community-based setting in Bengaluru [22]. Rani et al. [40] investigated the relationship between household food insecurity and mental health problems among teenage girls living in urban slums in Varanasi [40]. As discussed above, several studies have confirmed the positive association between food insecurity and depression. Some possible mechanisms have been discussed for the positive relationship between food insecurity and depression. The most common notion is that food insecurities may lead to depression due to stress about finding sufficient food resources [15].

As expected [39], our study noted a higher likelihood of depression among females than in males. Gender disparities in depression may stem from differences in behavioural and socio-economic factors, including diet, smoking, alcohol drinking, and education [44]. Also, higher depression among older females could partially be explained by postmenopausal depression and anxiety [2]. Studies unanimously reported that women tend to live longer than men but are expected to report being in worse health than men worldwide [34,35], including higher depression levels [30]. However insignificant, results noted a higher likelihood of food insecurity and a lower likelihood of depression among the elderly as they progress in their age group. Older adults are more likely to accept their ill-health as a sign of aging process and are more likely to remain silent on their cognitive status [6].

In line with previous studies in Indian [4,38] and other settings [46], this study also noted lower odds of depression among older adults reporting good self-rated health (SRH). The relationship between SRH and depression can be bidirectional, where depression can also lead to poor SRH [16]. Like previous studies [1,4], this study noticed lower odds of depression among older adults who were independent for ADL functions. It is understood that those who are independent for ADL functions are more likely to enjoy their life and more open to embracing life's challenges, leading to improved quality of life by reducing the risk of depression [32].

Unlike several previous studies in Indian [4] and different settings, this study noted that the odds of depression among older adults were higher among those with higher education. Those with higher education may likely be indulged in some kind of work and, as a result, may be less physically active. Physical activeness and depression have a well-known association, and studies have concluded that physical inactivity leads to depression [4,11]. In agreement with previous studies in Indian settings [4], this study noted a higher likelihood of depression among never-married older adults. However, a study in the community setting in India had noted otherwise results where depression was higher among married older adults than their unmarried counterparts [5].

## 5 Limitations and strengths of the study

Findings from this study must be considered within the context of several limitations. Although the data highlight the relationship between food insecurity and depression among the older population, one cannot make causal assumptions given that this is cross-sectional data. Data on food insecurity was self-reported, and respondents might have been embarrassed or uncomfortable discussing their food insecurity, leading to under-reporting [15]. Similarly, data on depression was also self-reported and might be under-reported due to the causes mentioned above. Despite the above limitations, the study has some sets of strengths too. This study used data having a large sample that is also country representative, and therefore, the findings can be generalized. Nevertheless, collapsing several variables into dichotomous form (Yes/No) might have led to losing some of the in-depth information in the data.

## 6 Conclusion

The present study highlights the importance of food security in maintaining mental health outcomes among older adults. The study explicitly noted a higher likelihood of depression among those having food insecurity. However, further study exploring the association with concrete evidence through a longitudinal approach may benefit the policy-makers. Further, the study found a higher likelihood of depression among those reporting poor self-rated health, not independent for ADL and IADL, female gender, never married older population, and highly educated older population. The study findings can be channelized for some policy discourse. It is important to integrate mental health with food insecurity. Providing food assistance to older adults may improve the mental health of such population. Since self-rated health and functional limitations were other significant predictors of depression among older adults, setting up geriatric healthcare clinics within Primary Health Centre (PHC) would improve older adults' overall quality of life.

## Financial disclosure statement

None of the authors received any financial support during preparation of this manuscript.

## Declaration of Competing Interest

None declared.

## References

[1] Ahmad N.A., Mohamad Kasim N., Mahmud N.A., Mohd Yusof Y., Othman S., Chan Y.Y. (2017). Prevalence and determinants of disability among adults in Malaysia: results from the National Health and Morbidity Survey (NHMS) 2015. BMC Public Health.

[2] Albert P.R. (2015). Why is depression more prevalent in women?. J Psychiatry Neurosci JPN.

[3] Andresen E.M., Malmgren J.A., Carter W.B., Patrick D.L. (1994). Screening for depression in well older adults: Evaluation of a short form of the CES-D. Am J Prev Med.

[4] Bhandari P., Paswan B. (2021). Lifestyle behaviours and mental health outcomes of elderly: modification of socio-economic and physical health effects. Ageing Int.

[5] Bincy K., Logaraj M., Ramraj B. (2021). Depression and its associated factors among the older adults in rural, Tamilnadu, India. Clin Epidemiol Glob Health.

[6] Bishwajit G., Kota K., Buh A., Yaya S. (2020). Self-reported food insecurity and depression among the older population in South Africa. Psych.

[7] Blue Bird Jernigan V., Wetherill M.S., Hearod J., Jacob T., Salvatore A.L., Cannady T. (2017). Food insecurity and chronic diseases among American Indians in Rural Oklahoma: the THRIVE study. Am J Public Health.

[8] Carruth L., Mendenhall E. (2019). “Wasting away”: diabetes, food insecurity, and medical insecurity in the Somali Region of Ethiopia. Soc Sci Med.

[9] Chatterjee N., Fernandes G., Hernandez M. (2012). Food insecurity in urban poor households in Mumbai, India. Food Secur.

[10] Chauhan S., Arokiasamy P. (2018). India’s demographic dividend: state-wise perspective. J Soc Econ Dev.

[11] Chauhan S., Srivastava S., Kumar P., Patel R., Dhillon P. (2020). Interaction of substance use with physical activity and its effect on depressive symptoms among adolescents. J Subst Abus.

[12] Chilton M., Booth S. (2007). Hunger of the body and hunger of the mind: African American Women’s perceptions of food insecurity, health and violence. J Nutr Educ Behav.

[13] Chinnakali P., Upadhyay R.P., Shokeen D., Singh K., Kaur M., Singh A.K. (2014). Prevalence of household-level food insecurity and its determinants in an urban resettlement Colony in North India. J Health Popul Nutr.

[14] Cole S.M., Tembo G. (2011). The effect of food insecurity on mental health: Panel evidence from rural Zambia. Soc Sci Med.

[15] Davey-Rothwell M.A., Flamm L.J., Kassa H.T., Latkin C.A. (2014). Food insecurity and depressive symptoms: comparison of drug using and nondrug-using women at risk for HIV. J Community Psychol.

[16] Falk H., Skoog I., Johansson L., Guerchet M., Mayston R., Hörder H. (2017). Self-rated health and its association with mortality in older adults in China, India and Latin America—A 10/66 Dementia Research Group study. Age Ageing.

[17] FAO (1996). http://www.fao.org/3/w3613e/w3613e00.htm.

[18] FAO I. (2017). Meeting the 2015 International Hunger Targets: Taking Stock of Uneven Progress.

[19] Giridhar G., Sathyanarayana K.M., Kumar S., James K.S. (2016). https://www.cambridge.org/core/journals/ageing-and-society/article/abs/g-giridhar-k-m-sathyanarayana-sanjay-kumar-k-s-james-and-moneer-alam-eds-population-ageing-in-india-cambridge-university-press-cambridge-2014-hardback-250-pp-isbn-13-9781107073326/32DB1F16224E999419B8BC4A2C8D41BA.

[20] Gundersen C., Ziliak J.P. (2015). Food insecurity and health outcomes. Health Aff.

[21] Hadley C., Tessema F., Muluneh A.T. (2012). Household food insecurity and caregiver distress: equal threats to child nutritional status?. Am J Hum Biol.

[22] Heylen E., Panicker S.T., Chandy S., Steward W.T., Ekstrand M.L. (2015). Food insecurity and its relation to psychological well-being among South Indian people living with HIV. AIDS Behav.

[23] Huddleston-Casas C., Charnigo R., Simmons L.A. (2009). Food insecurity and maternal depression in rural, low-income families: a longitudinal investigation. Public Health Nutr.

[24] Jones A.D. (2017). Food insecurity and mental health status: a global analysis of 149 countries. Am J Prev Med.

[25] Kaiser L., Baumrind N., Dumbauld S. (2007). Who is food-insecure in California? Findings from the California Women’s Health Survey, 2004. Public Health Nutr.

[26] Katz S., Ford A.B., Moskowitz R.W., Jackson B.A., Jaffe M.W. (1963). Studies of illness in the aged: the index of ADL: a standardized measure of biological and psychosocial function. JAMA.

[27] The Longitudinal Ageing Study in India (LASI) 2017-18 (2020).

[28] Lawton M.P., Brody E.M. (1969). Assessment of older people: self-maintaining and instrumental activities of daily living. Gerontologist.

[29] Leung C.W., Epel E.S., Willett W.C., Rimm E.B., Laraia B.A. (2015). Household food insecurity is positively associated with depression among low-income supplemental nutrition assistance program participants and income-eligible nonparticipants. J Nutr.

[30] McDonough P., Walters V. (2001). Gender and health: reassessing patterns and explanations. Soc Sci Med.

[31] Melchior M., Chastang J.-F., Falissard B., Galéra C., Tremblay R.E., Côté S.M. (2012). Food insecurity and children’s mental health: a prospective birth cohort study. PLoS One.

[32] Na L., Streim J.E. (2017). Psychosocial well-being associated with activity of daily living stages among community-dwelling older adults. Gerontol Geriatr Med.

[33] Nakulan A., Sumesh T.P., Kumar S., Rejani P.P., Shaji K.S. (2015). Prevalence and risk factors for depression among community resident older people in Kerala. Indian J Psychiatry.

[34] Oksuzyan A., Juel K., Vaupel J.W., Christensen K. (2008). Men: Good health and high mortality. Sex differences in health and aging. Aging Clin Exp Res.

[35] Oksuzyan A., Singh P.K., Christensen K., Jasilionis D. (2018). A cross-national study of the gender gap in health among older adults in India and China: similarities and disparities. Gerontologist.

[36] Patel R., Chauhan S. (2020). Gender differential in health care utilisation in India. Clin Epidemiol Glob Health.

[37] Patel R., Chauhan S., Chaurasiya D., Kumar S., Paswan B. (2019). Role and impact of social capital on health of older adult in India. Indian J Sol Res.

[38] Patel R., Marbaniang S.P., Srivastava S., Kumar P., Chauhan S., Simon D.J. (2021). Gender differential in low psychological health and low subjective well-being among older adults in India: With special focus on childless older adults. Plos One.

[39] Pilania M., Yadav V., Bairwa M., Behera P., Gupta S.D., Khurana H. (2019). Prevalence of depression among the elderly (60 years and above) population in India, 1997–2016: a systematic review and meta-analysis. BMC Public Health.

[40] Rani D., Singh J.K., Acharya D., Paudel R., Lee K., Singh S.P. (2018). Household food insecurity and mental health among teenage girls living in urban slums in Varanasi, India: a cross-sectional study. Int J Environ Res Public Health.

[41] Silverman J., Krieger J., Kiefer M., Hebert P., Robinson J., Nelson K. (2015). The relationship between food insecurity and depression, diabetes distress and medication adherence among low-income patients with poorly-controlled diabetes. J Gen Intern Med.

[42] Smith L., Shin J.I., McDermott D., Jacob L., Barnett Y., López-Sánchez G.F. (2021). Association between food insecurity and depression among older adults from low- and middle-income countries. Depress Anxiety.

[43] Suryanarayana M.H., Silva D. (2007). Is targeting the poor a penalty on the food insecure? Poverty and food insecurity in India. J Hum Dev.

[44] Verplaetse T.L., Smith P.H., Pittman B.P., Mazure C.M., McKee S.A. (2016). Associations of gender, smoking, and stress with transitions in major depression diagnoses. Yale J Biol Med.

[45] Weaver L.J., Fasel C.B. (2018). A systematic review of the literature on the relationships between chronic diseases and food insecurity. Food Nutr Sci.

[46] Wuorela M., Lavonius S., Salminen M., Vahlberg T., Viitanen M., Viikari L. (2020). Self-rated health and objective health status as predictors of all-cause mortality among older people: A prospective study with a 5-, 10-, and 27-year follow-up. BMC Geriatr.

